# ObReco-2: Two-step validation of a tool to assess memory deficits using 360° videos

**DOI:** 10.3389/fnagi.2022.875748

**Published:** 2022-07-28

**Authors:** Francesca Bruni, Valentina Mancuso, Chiara Stramba-Badiale, Luca Greci, Marco Cavallo, Francesca Borghesi, Giuseppe Riva, Pietro Cipresso, Marco Stramba-Badiale, Elisa Pedroli

**Affiliations:** ^1^Faculty of Psychology, eCampus University, Novedrate, Italy; ^2^Applied Technology for Neuropsychology Lab, IRCCS Istituto Auxologico Italiano, Milan, Italy; ^3^Institute of Intelligent Industrial Technologies and System for Advanced Manufacturing, Milan, Italy; ^4^Human Technology Lab, Catholic University of the Sacred Heart, Milan, Italy; ^5^Department of Psychology, University of Turin, Turin, Italy; ^6^Department of Geriatrics and Cardiovascular Medicine, IRCCS Istituto Auxologico Italiano, Milan, Italy

**Keywords:** memory, neuropsychological assessment, 360° video, virtual reality, object recognition, neuroscience

## Abstract

Traditional neuropsychological evaluations are usually carried out using psychometric paper and pencil tests. Nevertheless, there is a continuous discussion concerning their efficacy to capture life-like abilities. The introduction of new technologies, such as Virtual Reality (VR) and 360° spherical photos and videos, has improved the ecological validity of the neuropsychological assessment. The possibility of simulating realistic environments and situations allows clinicians to evaluate patients in realistic activities. Moreover, 360° photos and videos seem to provide higher levels of graphical realism and technical user-friendliness compared to standard VR, regardless of their limitations in terms of interactivity. We developed a novel 360° tool, ObReco-2 (Object Recognition version 2), for the assessment of visual memory which simulates a daily situation in a virtual house. More precisely, patients are asked to memorize some objects that need to be moved for a relocation. After this phase, they are asked to recall them after 15 min and later to recognize them in the same environment. Here we present a first study about the usability of ObReco-2, and a second one exploring its clinical efficacy and updated usability data. We focused on Free Recall and Recognition scores, comparing the performances obtained by the participants in the standard and the 360° test. The preliminary results support the use of 360° technology for enhancing the ecological value of standard memory assessment tests.

## Introduction

Recently, the debate regarding the ecological validity of the measures typically employed for the assessment of cognitive domains seems to be an open-ended question in the neuropsychological field. Ecological validity refers to the degree of association between what is observed during neuropsychological testing and real-life activities, i.e., the ability of paper and pencil tests to predict real-life functioning (Sbordone, 1996). Despite the widespread use of paper and pencil tests, their ability to predict patients’ skills in real-life circumstances could be limited (Neguţ et al., 2016; Rizzo and Koenig, 2017). One of the main issues is that patients may not show deficits in the clinical setting but at the same time report some difficulties in everyday situations or vice versa (Chaytor and Schmitter-Edgecombe, 2003; Mondini et al., 2016). Indeed, during the clinical evaluation patients are required to carry out various behavioral and cognitive activities in a controlled setting which may not always predict their functioning in a real-life situation. Therefore, it is worth considering the debate over the efficacy of many traditional tests assuming a more function-based approach rather than a construct-based one (Parsons, 2015; Parsons et al., 2017; Serino and Repetto, 2018). A construct-based approach starts from a solid theoretical paradigm assessing abstract constructs without an explicit interest in predicting real-life functional abilities. On the other hand, a function-based approach arises from direct observations of patients’ performance in real-life contexts to guarantee a more ecological assessment (Sbordone, 1996; Parsons, 2015; Parsons et al., 2017). The Rivermead Behavioural Memory Test (RBMT) is the most well-known example of this approach for memory assessment (Wilson et al., 1989). It includes a series of daily-life tasks such as locating personal objects, remembering an appointment, recalling an itinerary, etc.

In recent years, technologies might be considered promising realities in accomplishing and improving ecological validity, sensitivity, and specificity of traditional assessment methods. Among these, virtual reality (VR) emerges as a suitable possibility in neuropsychological assessment. This technology can be employed to develop highly ecological and controlled environments resembling the real-life contexts in which patients’ daily activities usually take place (Riva and Mantovani, 2014; Neguţ et al., 2016; Riva et al., 2019). It thus can allow researchers and clinicians to measure cognitive and motor abilities in naturalistic environments, obtaining better prognostic indexes of real-life functioning in a safe and controlled situation. This approach has been widely used in the medical and neuropsychological field to assess and treat different pathologies such as traumatic brain injury (Aida et al., 2018; Alashram et al., 2019) and post-stroke (Saposnik and Levin, 2011; Laver et al., 2017). Moreover, it has been revealed promising for balance deficits (Allain et al., 2014) and memory impairments (Matheis et al., 2007; Ouellet et al., 2018; Serino and Repetto, 2018). More specifically, memory interventions included several tasks in which patients were required to perform some activities while navigating in the 3D environments (i.e., office and supermarket) (Matheis et al., 2007; Ouellet et al., 2018; Serino and Repetto, 2018). The employment of 360° immersive photos and videos is a growing declination of VR technology that may offer promising outcomes (Serino and Repetto, 2018; Realdon et al., 2019; Ventura et al., 2019). They are spherical videos or photos captured by an omnidirectional camera. As previously mentioned, this method has greater benefits than graphic-based VR as it can capture the real environment, providing a high level of visual realism that can increase participant engagement. Moreover, this technology is inexpensive and easy-to-use (Bohil et al., 2011). Furthermore, the user-friendly design makes 360° technologies more suitable for the assessment of patients with mild to severe impairments (Sbordone, 1996; Realdon et al., 2019) who may have some difficulties interacting with more sophisticated devices.

The present study aims to test a 360° technology for memory assessment compared to a traditional paper and pencil test included in the RBMT-III (Wilson et al., 2008; Beschin and Urbano, 2013). Based on promising results from an earlier pilot study showing the feasibility of a 360° memory assessment (Pieri et al., 2021), we improved technology using higher-level equipment to design ObReco-2 (Object Recognition version 2). Firstly, we present the results of a usability study (Study 1), and then the results of the clinical efficacy along with updated usability data (Study 2).

## Study 1 (usability study)

### Materials and methods

#### Participants

For the usability assessment, participants were enrolled among the patients and outpatients of the Department of Medical Rehabilitation of Istituto Auxologico Italiano in Milan. They were volunteers aged over 60 (without maximum age limitation), with a normal or corrected-to-normal vision. Exclusion criteria were: (i) invalidating internist, psychiatric, neurological conditions which could affect the usability of the task; (ii) cognitive impairments certifiable by a score at the Mini-Mental State Examination (MMSE) Italian version (Measso et al., 1993; Magni et al., 1996) lower than 24 points. The resulting sample included 10 participants (6 females and 4 males), with a mean age of 75.5 (SD = 5.36) and a mean of 12.3 (SD = 3.89) years of education. All the subjects’ demographic data and MMSE scores are reported in Table 1. Before the usability session, all participants signed the informant consent. The study received ethical approval from the Ethical Committee of the Istituto Auxologico Italiano.

**TABLE 1:** Demographic data and mini-mental state examination scores.

Descriptives			
	Years	Education	MMSE
Mean	75.5	12.3	25.8
Standard deviation	5.36	3.89	1.47
Min	68	4	23.3
Max	84	18	28.0

#### Materials

Files were recorded in a real environment using the Insta 360 ONE X, an omnidirectional video camera that can record spherical photos and videos with a resolution respectively of 608 × 3040 and 5.760 × 2.880 pixels. We combined all photos and videos into a single interactive experience, using the InstaVR software©. The result consists of an application deliverable via smartphone that may be experienced using a Cardboard, which allows the user to navigate within this immersive 360° scenario. In particular, the application was provided via an InstaVR link on the smartphone which was inserted into the Cardboard to show the environment.

#### Procedure

For this study participants were examined in two sessions at a maximum of 2 days apart. In the first session, the MMSE was administered to quantify the general cognitive state of the patients. The second phase of the study consisted of a usability study employing cardboard (Daydream view©). Usability is a key factor that needs to be evaluated when employing new technologies. It can be defined as the degree to which a user can utilize a given system to achieve specific goals effectively, efficiently, and satisfactorily. Usability test allows the clinicians to identify obstacles and facilitators, develop appropriate tasks for the target, define the usability criteria and test its clinical use (Tuena et al., 2020). During the usability session, all the participants were sitting on a turning chair, to freely explore the 360° virtual environments using the cardboard.

#### User experience measures

In the present study, the usability has been assessed using the System Usability Scale (SUS) (Brooke, 2020), the Senior Technology Acceptance Model (STAM) (Chen and Lou, 2020), the thinking aloud protocol (TAP) (Lewis, 1982), and the Independent Television Commission Sense of Presence Inventory (ITC-SOPI) to assess the cybersickness (Lessiter et al., 2001). The SUS (Brooke, 2020) is a “quick and easy-to-use” questionnaire which includes ten items describing the user’s feeling concerning the interaction with the technology. For each of these answers, the participants need to define their degree of agreement using a 5-point Likert scale ranging from “Strongly Agree” to “Strongly Disagree”. The final score ranges from 0 (lack of usability) to 100 (optimal usability). The STAM is a 13-items questionnaire that analyzes four components of the STAM: attitude through technologies, perception of control, anxiety related to technologies, and general health conditions (Chen and Lou, 2020). The TAP (Lewis, 1982) is a qualitative technique that is generally administered to test the usability of new technology. Subjects are asked to express their opinion regarding the technology employment and criticism while performing the task. The observer, on the other hand, is asked to take notes of participants’ observations and concerns without attempting to interpret their actions and words. All the verbalizations are transcribed and analyzed to develop the formal usability report. The ITC-SOPI (Lessiter et al., 2001) is a questionnaire that includes 44 items addressing the individual’s feelings after the VR experience. Participants are asked to determine their degree of agreement with each of these sentences using a 5-point Likert scale ranging from “Strongly Agree” to “Strongly Disagree”. The ITC-SOPI includes four subscales: Sense of Physical Space (19 items), Engagement (13 items), Ecological Validity (5 items), and Negative Effects (6 items).

#### ObReco-2

**FIGURE 1:**
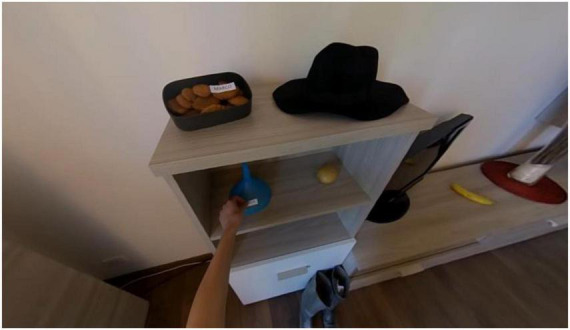
A screenshot showing the task presented during the encoding phase. Here the experimenter is labeling a target object.

**FIGURE 2:**
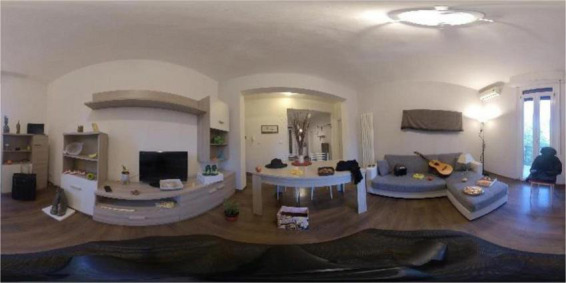
The panoramic photo of the room in which target objects are mixed with distractors.

ObReco-2 is a 360° task aimed to assess visual memory simulating a real-life situation in a daily setting. Users are immersed in a virtual living room, in which they are required to encode and then recall some target objects that have been relocated, as described in Bruni et al. (2022). The virtual interactive experience consists of a series of different phases:

(i) *Familiarization*. Patients who wear the headset find themselves immersed in a natural 360° landscape; here they have to explore the environment. The objective is to make the patient familiar with the technology and to detect possible side effects (i.e., cybersickness).
(ii) *Encoding*. On a black screen, the participants are first given a brief explanation of the context: Marco, who is living with other roommates, must move and he had to relocate all of his possessions, thus he labels them with his name. Participants experience a household setting, such as a living room, in which they can with a first-person perspective which is the one of the experimenter. This one moves about the room highlighting the 15 target items for 3 s each and attaching a tag bearing the name “Marco” to each one Figure 1). In the living room, there are also 15 other objects used as distractors. In this phase, participants are instructed to name all the targets.
(iii) *Interference*. Participants are asked to take off their headsets and complete non-verbal tasks15 for minutes.
(iv) *Free recall*. They are instructed to name as many objects from the encoding phase as they can.
(v) *Recognition*. Participants had to wear the headset once again for this last section. They are instructed to explore the prior living room (Figure 2), discover and name the target objects among all of the previous things and an unknown set of 15 distractor objects.

### Data analysis

We organized all the data collected in a Windows Excel sheet and we performed descriptive analyses of the usability questionnaires investigating users’ experience with technology.

### Results

The descriptive user experience (UX) measures are shown in Table 2.

**TABLE 2:** Descriptives of the user experience (UX) measures.

	SUS	STAM-a	STAM-c	STAM-anx	STAM-h	ITC-sp	ITC-e	ITC-ev	ITC-ne
Mean	69.3/100	6.10/10	7.13/10	5.60/10	8.58/10	2.94/5	3.33/5	3.96/5	1.87/5
Standard deviation	18.1	3.07	1.98	3.07	1.80	1.03	0.73	0.94	0.90
Min	40.0	1.00	3.25	1.00	4.60	1.18	1.77	2.00	1.00
Max	100	9.33	10.0	10.0	11.2	4.25	4.31	4.80	3.83

For each measure there are mean and the maximum available score, standard deviation and minimum (min) and maximum (max) score reported by participants.

SUS, System Usability Scale; CSQ; STAM-a, attitude through technologies subscale; STAM-c, Senior Technology Acceptance Model perception of control subscale; STAM-anx, Senior Technology Acceptance Model anxiety related to technologies subscale; STAM-h, Senior Technology Acceptance Mode health conditions subscale; ITC-sp, Independent Television Commission Sense of Presence Inventory-Sense of Physical Space subscale; ITC-e, Engagement subscale; ITC-ev, Ecological Validity subscale; ITC-ne, Negative Effects subscale.

**FIGURE 3:**
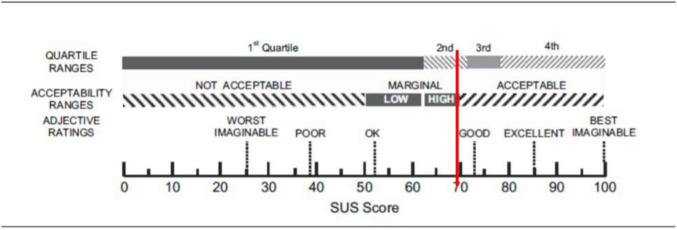
Graphical representation of the interpretation of system usability scale (SUS). The vertical line shows the position of the SUS mean score (69.3) obtained in study 1 according to the rating comparison scale provided by Bangor et al. (2009).

Starting from quantitative data, the mean score of the SUS is 69.3 (SD = 18.1). According to Bangor et al. (2009) this score indicates that ObReco-2 is placed in a marginal zone between *Ok* and a *Good level* of usability as shown in Figure 3.

The results of the STAM scale reveal that our sample has a positive attitude toward technology (M = 6.10/10; SD = 3.7), has good control/access to technological devices (M = 7,13/10; SD = 1.98), has a medium level of technology-related anxiety (M = 5.60/10; SD = 3.07), and considers themselves in good health conditions (M = 8.58/10; SD = 1.8). As shown by the ITC-SOPI sub-scale investigating negative effects, all subjects reported minimal side effects (M = 1.87; SD = 0.90) indicating that the use of ObReco-2 did not determine dizziness and cybersickness. Qualitative results of the thinking aloud protocol are shown in Table 3. It is structured as follows: (i) description of the task (1st column), (ii) problems encountered by patients (2nd column), (iii) some possible solution for those problems (3rd column), and (iv) number of patients that encountered problems (4th column). Overall, patients did not encounter problems using the cardboard. However, most patients reported difficulties in the encoding exercise in which they were required to explore the room following labels and naming Marco’s objects. Five patients had difficulty in exploring the environment. Four patients reported unclear images; one didn’t name all the objects and four people had difficulty finding the initial correct direction to follow the 360° video. Finally, one person reported nausea.

**TABLE 3:** Qualitative usability results of thinking aloud protocol.

Task	Problem	Solution	N.S.
**Use of cardboard**			
Wear cardboard	None	None	–
Remove cardboard	Sense of annoyance/sense of falling after removing the cardboard	Encourage the patient to keep his/her eyes open to avoid falling	1
**Instructions**			
Listening	None	None	–
Comprehension	None	None	–
**Familiarization**			
Listening	None	None	–
Comprehension	None	None	-
Execution	Blurry image	Improve the quality of VR video	1
**Encoding**			
Listening	None	None	–
Comprehension	None	None	–
Execution	Difficulty to explore the environment in an appropriate order	Improve instructions’ clarity	5
		Encourage to listen carefully the instructions	
	Unclear image	Improve the quality of images	4
	Name all the objects	Improve instructions’ clarity	1
	Difficulty to find the initial object labeled	Improve the instructions	4
	Nausea	Provide slower execution of the exercise	1
**Recognition**			
Listening	None	None	–
Comprehension	None	None	–
Execution	Dizziness	Provide slower execution of the exercise	1
	Recognizes many distractors caused by blurry image	Improve the quality of images	1

## Study 2 (usability study and clinical efficacy)

### Materials and methods

#### Participants

For this clinical efficacy and usability study, 20 patients were enrolled at the Department of Medical Rehabilitation of Istituto Auxologico Italiano in Milan. They were volunteers aged over 55, with a normal or corrected-to-normal vision. Exclusion criteria were (i) invalidating internist, psychiatric, or neurological conditions which could affect the task; (ii) cognitive impairments difficulties certifiable by a score at the MMSE Italian version lower than 24 points (Measso et al., 1993; Magni et al., 1996). Before the session, all participants signed the informant consent. The study received ethical approval from the Ethical Committee of the Istituto Auxologico Italiano. The sample was composed by 12 females and 8 males, divided in experimental group (ObReco-2–VR) (Mean age = 68.2 years, SD = 5.45, mean education = 12, SD = 4.45, 6 females) and control group (RBMT-III—paper and pencil) (Mean age = 69.7 years, SD = 7.63, mean education = 14.6, SD = 3.84, 6 females). All the subjects’ demographic data and MMSE scores are reported in Table 4. The two groups are comparable in age *t*(18) = 0.506, *p* = 0.619, in years of education t(18) = 1.400, *p* = 0.179 and MMSE *t*(18) = 0.506, *p* = 0.619.

**TABLE 4:** Demographic data and mini-mental state examination scores.

Descriptives						

	Years_PP	Education_PP	MMSE_PP	Years_VR	Education_VR	MMSE_VR
Mean	69.7	14.6	27.6	68.2	12.0	27.2
Standard deviation	7.63	3.84	1.79	5.45	4.45	1.74
Min	57	8	25.5	59	5	24.7
Max	81	18	30.0	75	18	30.0

PP, paper and pencil group; VR, virtual reality group.

#### Materials

Improving VR experience, we implemented all the previously collected files into a single interactive experience, using Unity3D©. The result consists of an interactive application deliverable through a head-mounted display (HMD), which allows the user to navigate and interact within the immersive 360° scenario. The application was downloaded and installed directly on an Oculus Quest-2© HMD to be used without any restrictions. Thanks to the most advanced functionalities, participants can have major interactivity with the environment, without the continuous intervention of the experimenter. In the first usability study, subjects were limited in their interaction with the environment; different links were provided to them, corresponding to the different parts of the ObReco-2. Indeed, at the end of each task, the experimenter required them to remove the Cardboard in order to provide the next link. Here all tasks were provided in a unique VR experience.

#### Procedure

The study involved randomized between-subject data collection. Each participant performed two sessions, that lasted about one hour, at a maximum of two days apart. In the first session, a neuropsychological assessment was performed, then participants were randomly assigned to different conditions: the traditional paper and pencil tests (RBMT-III Italian Version) and the experimental one (ObReco-2). During the 360° session all the participants were sitting on a turning chair, to freely explore the virtual environments using an Oculus Quest-2© HMD.

#### Neuropsychological assessment

The neuropsychological evaluation included the MMSE, the Frontal Assessment Battery (FAB) Italian Version (Appollonio et al., 2005), the Babcock Story Recall Test (BSRT) Italian Version (Spinnler and Tognoni, 1987), the Rey Auditory Learning test (RAVLT) (Carlesimo et al., 1996), the Tower of London (ToL) (Allamanno et al., 1987), Attentive matrices (Spinnler and Tognoni, 1987), test exploring Constructive Apraxia (Arrigoni and de Renzi, 1964), Trail Making Test (TMT) (Amodio et al., 2002) and Raven’s progressive matrices (Caffarra et al., 2003). Moreover, participants performed the Picture Recognition sub-test included in the RBMT-III Italian Version (Beschin and Urbano, 2013). The Picture Recognition is a sub-test of the RBMT-III. It is divided into two parts: the encoding and the recognition phases. During the encoding, the patient is asked to see a set of 15 pictures representing common animate and inanimate objects (e.g., a clock, a chicken) and to recognize and name each one of them. In the recognition phase, the participant is asked to observe a set of 30 pictures including target items (i.e., the 15 pictures presented in the Encoding Phase) and distractors (i.e., 15 pictures not included in the Encoding Phase): for each of these, the patient is asked to answer yes if the picture was presented previously or no if it was not. During the Recognition task, several measures are collected: the HR (the proportion of yes responses to old items) the False Alarm Rate (the proportion of yes responses to distractors), and the False Alarm Unknown (the proportion of yes responses to unknown distractors) (Snodgrass and Corwin, 1988). The raw score obtained in the sub-test is the number of pictures correctly recognized. Moreover, before the Recognition Phase, we included a Free Recall task, in which the patient was required to recall every object he/she could from those presented in the encoding phase. The raw score is defined by the number of objects correctly reported.

### Data analysis

All the analyses were performed using Jamovi Software (The jamovi project, 2021). After having collected all the data in a Windows Excel sheet, we computed different indexes for both the RBMT-III and ObReco-2. In particular, for the recognition tasks, we computed three different scores: the HR, (the proportion of yes responses to targets), the False Alarm Rate (the proporton of yes responses to distractors), and the False Alarm “unknown” (the proportion of yes responses to unknown distractors, i.e., objects that were not included in the encoding phase). Then, we performed Mann–Whitney U tests to compare the free recall and recognition scores in both RBMT-III and 360° modalities, investigating the statistically significant differences in the two performances. We also performed correlation analyses to investigate relationships between neuropsychological examinations and memory indices from RBMT-III and ObReco-2. At last, we performed descriptive analyses of the usability questionnaires and then we compared (using Mann–Whitney U) usability scores of study 1, in which participants used cardboard, and study 2 where otherwise they used an Oculus Quest-2.

### Results

#### Usability

**FIGURE 4:**
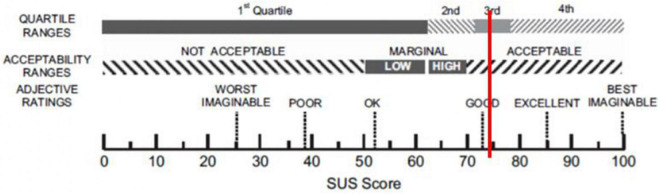
Graphical representation of the interpretation of system usability scale (SUS). The vertical line shows the position of the SUS mean score (74) obtained by the Oculus Quest according to the rating comparison scale provided by Bangor et al. (2009).

Starting from quantitative data, the mean score of the SUS is 74 (SD = 14.7). According to Bangor et al. (2009) this score indicates that ObReco-2 is placed in a *Good Level* of usability as shown in Figure 4.

The results of the STAM scale reveal that our sample has a positive attitude toward technology (M = 6.81/10/10; SD = 2.98), has good control/access to technological devices (M = 7.39/10; SD = 1.66), has a medium level of technology-related anxiety (M = 6/10; SD = 2.81), and considers themselves in good health conditions (M = 7.62/10; SD = 1.31). As shown by the ITC-SOPI sub-scale investigating negative effects, all subjects reported minimal side effects (M = 1.90; SD = 1.79) indicating that the use of ObReco-2 did not determine dizziness and cybersickness. The descriptive of UX measures are shown in Table 5. Considering qualitative results of the Thinking Aloud Protocol, a limited number of patients referred to similar problems to those observed in the cardboard’s group: they mentioned blurry images and difficulty to identify where the labels are immediately when the task started. At last, we compared the usability scores of study 1 (Cardboard) and study 2 (Oculus Quest-2). The results of the independent *t*-test reveal non-statistically significant differences suggesting that both cardboard and Oculus Quest-2 are easy-to-use technologies.

**TABLE 5:** Desciptives of the user experience (UX) measures of the study 2.

	SUS	STAM-a	STAM-c	STAM-anx	STAM-h	ITC-sp	ITC-e	ITC-ev	ITC-ne
Mean	74/100	6.81/10	7.39/10	6/10	7.62/10	3.43/5	3.87/5	4.12/5	1.90/5
Standard deviation	14.73	2.98	1.66	2.81	1.32	0.86	0.78	0.71	1.79
Min	55.00	1.00	3.5	2.00	5.60	1.80	2.30	3.00	1.00
Max	90.00	10.00	9.50	10.00	9.20	4.60	4.80	5.00	6.80

For each measure there are mean and the maximum available score, standard deviation and minimum (min) and maximum (max) score reported by participants.

SUS, System Usability Scale; CSQ; STAM-a, attitude through technologies subscale; STAM-c, Senior Technology Acceptance Model perception of control subscale; STAM-anx, Senior Technology Acceptance Model anxiety related to technologies subscale; STAM-h, Senior Technology Acceptance Mode health conditions subscale; ITC-sp, Independent Television Commission Sense of Presence Inventory-Sense of Physical Space subscale; ITC-e, Engagement subscale; ITC-ev, Ecological Validity subscale; ITC-ne, Negative Effects subscale.

### Clinical efficacy

The descriptives of the accuracy on free recall and recognition tasks performances of two groups are presented in Table 6. The results indicate that for the free recall tasks, participants performed better after ObReco-2 than RBMT-III in terms of the number of targets correctly recalled although the difference is not statistically significant (U = 39.0, *p* = 0.416). Concerning the recognition indexes, participants recognized more objects after the standard presentation compared to the 360° one, and the observed difference is statistically significant (U = 21.5, *p* = 0.029). We also performed a statistical analysis to investigate correlations between neuropsychological examinations and memory indices from RBMT and ObReco-2. In the control group, results show a statistically significant correlation between FAB and RBMT Recognition (HR) (*r* = 0.687, *p* = 0.028). None of the other neuropsychological tests correlates with RBMT. On the other hand, in the experimental group ObReco-2 scores correlate with AM (*r* = 0.642, *p* = 0.045) and delayed RAVLT (*r* = 0.645, *p* = 0.044).

**TABLE 6:** The table shows the descriptives of the accuracy obtained by the participants in the free recall tasks (FR) and the correct objects identified in the recognition task hit rate (HR) in the standard Rivermead Behavioural Memory Test (RBMT) and virtual reality (VR) (ObReco) conditions. The last column indicates the false alarms (FAR) i.e., the yes responses to the wrong items.

Descriptives					

	RBMT_FR	OBRECO_FR	RBMT_HR	OBRECO_HR	OBRECO_FAR
Mean	5.30	6.20	11.1	9.20	1.30
Standard deviation	2.45	2.10	1.52	1.81	0.675

## Discussion

The ongoing scientific debate about the ecological validity of classical assessment encourages the implementation of VR in neuropsychological assessment (Chaytor and Schmitter-Edgecombe, 2003; Parsons, 2015; Neguţ et al., 2016). Based on this rationale, we aimed to design an assessment tool that used naturalistic and life-like situations; we decided to develop an application using 360°contents which allow a more ecological performance rather than computer-generated VR (Serino and Repetto, 2018). The results are promising: patients were satisfied with the application and they expressed interest in trying a new assessment methodology. They were fascinated by the exploration of a virtual environment, and they reported enjoyment in performing exercises in this innovative way. Furthermore, results revealed minimal negative effects while wearing the cardboard. Only a small number of them experienced dizziness or sickness as a possible collateral effect. However, considering the experience with the cardboard, it was limited: patients could not interact directly with the environment due to technical restrictions. Every phase of the task required the experimenter’s intervention and thus the experience of the users was not continuative. To overcome these limitations, we designed and ameliorated this task by employing an advanced technology: the Oculus Quest-2. Results of the usability scales revealed that ObReco-2 is *Acceptable* and has a *Good Level* of usability (Bangor et al., 2009). It means that the product was judged “goodness” by users. Positive outcomes came also from the results of the STAM scale (Chen and Lou, 2020), confirming users’ acceptance and usage. It means that the four key factors identified by the model, *performance expectation*, *effort expectation*, *anxiety related to technology*, *and facilitating conditions* (health condition) predict the intent to use the proposed tool. Technology acceptance is the perception of attitudes and behavioral intent to use technology, and it is a major predictor of technology adoption and usage. In support of this, the Negative Effects scale indicates minimal dizziness and cybersickness. This result could be explained by the minimal movement required in the VR environment and the limited duration of the VR exposure (about 10 min). Nevertheless, some technical problems were reported by the TAP (Lewis, 1982) in both usability studies, possible solutions for the main described issues could be improving the clarity of the instructions, adding a more specific training phase, improving the quality of images, and suggesting a slower execution of the exercise. Although the two study groups were different and we couldn’t compare the two devices, the UX measures of both, cardboard and Oculus, do not seem to differ. On one hand, the lack of interactivity of the cardboard could have been experienced as an advantage instead of a limit for a sample of old people who don’t have proper skills with technologies. In this way, cardboard may be managed more simply and quickly. On the other hand, the experience provided by the Oculus Quest-2 in terms of immersivity, sense of presence, and engagement guarantee better immersive quality and better VR experience which explain the high scores of acceptability for this device.

A further purpose of this exploratory study was to test the efficacy of 360° technology in neuropsychological memory assessment. Considering previous results from literature (Serino et al., 2017; Realdon et al., 2019) we expected to find some correlations between memory performances in the standard sub-test of the RBMT-III and the ObReco-2. The results indicated that participants obtained higher scores on the free recall tasks in the virtual conditions, showing a better trend performance after the ObReco-2, although this difference is not statistically significant. This trend could be explained by other factors including engagement and interaction provided by the VR experience. In fact, in agreement with previous studies (Robertson et al., 2016; Makowski et al., 2017), a higher level of immersivity and realism leads to better memory encoding. The photorealism of 360° environments may have elicited a visual memory encoding similar to that seen in everyday life, resulting in the greater visual encoding of stimuli, easier recall of items, and increased ecological validity of the evaluation technique. Overall, these results are consistent with those found by Pieri et al., which used a minor number of target objects to be remembered (Pieri et al., 2021). For what concerns the recognition performance, the pattern of results is inverted: participants showed high levels of accuracy in both conditions but performed significantly better in the RBMT-III condition. These results could be explained by analyzing the participants’ qualitative reports. They described difficulties in recognizing objects during the encoding phase in the virtual task, due to the low quality of the video. Future studies could introduce a preliminary naming test to verify this condition. Moreover, while in the RBMT-III participants had to encode one object per time, in the virtual task all the target objects were shown in the environment at the same time. This could have prevented them to focus their attention singularly on each object, although this condition is the most similar to real-life situations. This complexity reflects the daily routine in which ecological patterns require actively exploring the space to discriminate the target items from the distractors. This may have allowed a slightly more sensitive and ecological assessment of recognition memory when compared to the RBMT-III condition.

## Limitation and conclusion

The present work is not exempt from limitations. First, the sample is restricted in its size and representativity. We primarily focused on the features of the technology, but further studies must include a larger sample size with different demographic characteristics. The second gap regards the technological equipment, currently, the 360° devices market offers much higher-quality omnidirectional cameras (e.g., Insta360 Pro 2©) which can provide a higher-quality of images and a higher ecological value to the obtained measures. Then, another limitation refers to the difference between samples of study 1 and study 2 in terms of MMSE scores.

Even with its limitations, these findings show the feasibility of 360°-VR assessment, thus encouraging the implementation of this technology in the development of ecological tests for memory evaluation. Based on these assumptions, future studies are needed to develop and validate standardized applications for the assessment of different cognitive domains but also different memories, for example, semantic or autobiographical. Further works are also required to clarify which advantages and disadvantages characterize VR, to improve the design of 360° experiences, and to investigate cognitive assessment using the innovative proposed tool in different populations.

## Data availability statement

The raw data supporting the conclusions of this article will be made available by the authors, without undue reservation.

## Ethics statement

The studies involving human participants were reviewed and approved by Ethical Commitee-IRCCS Istituto Auxologico Italiano. The patients/participants provided their written informed consent to participate in this study.

## Author contributions

FBr designed the protocol and write the first draft. CS-B and VM participated in the design and contributed to the data collection. LG developed the environments. PC, EP, and FBo analyzed the data. MC and MS-B provided the required revisions. GR supervised and contributed to the reviewed version of the manuscript. PC, MS-B, and EP have supervised the study. All authors have approved the final version of the manuscript. All authors contributed to the article and approved the submitted version.
